# The gut–liver axis in immune remodeling of hepatic cirrhosis

**DOI:** 10.3389/fimmu.2022.946628

**Published:** 2022-08-08

**Authors:** Huayu Guan, Xiang Zhang, Ming Kuang, Jun Yu

**Affiliations:** ^1^ Department of Medicine and Therapeutics, State Key Laboratory of Digestive Disease, Institute of Digestive Disease, The Chinese University of Hong Kong, Hong Kong, Hong Kong SAR, China; ^2^ Department of Liver Surgery, Center of Hepato-Pancreato-Biliary Surgery, The First Affiliated Hospital, Sun Yat-sen University, Guangzhou, China

**Keywords:** gut-liver axis, microbiota, immune, gut microbiome, liver cirrhosis, immune homeostasis, immune remodeling

## Abstract

In healthy settings, the gut–liver axis allows host–microbiota communications and mediates immune homeostasis through bidirectional regulation. Meanwhile, in diseases, gut dysbiosis, combined with an impaired intestinal barrier, introduces pathogens and their toxic metabolites into the system, causing massive immune alternations in the liver and other extrahepatic organs. Accumulating evidence suggests that these immune changes are associated with the progression of many liver diseases, especially hepatic cirrhosis. Pathogen-associated molecular patterns that originated from gut microbes directly stimulate hepatocytes and liver immune cells through different pattern recognition receptors, a process further facilitated by damage-associated molecular patterns released from injured hepatocytes. Hepatic stellate cells, along with other immune cells, contribute to this proinflammatory and profibrogenic transformation. Moreover, cirrhosis-associated immune dysfunction, an imbalanced immune status characterized by systemic inflammation and immune deficiency, is linked to gut dysbiosis. Though the systemic inflammation hypothesis starts to link gut dysbiosis to decompensated cirrhosis from a clinical perspective, a clearer demonstration is still needed for the role of the gut–liver–immune axis in cirrhosis progression. This review discusses the different immune states of the gut–liver axis in both healthy and cirrhotic settings and, more importantly, summarizes the current evidence about how microbiota-derived immune remodeling contributes to the progression of hepatic cirrhosis *via* the gut–liver axis.

## Introduction

The gut–liver axis is the bidirectional communication between the intestine, its microbiota, and the liver. While receiving nutrient-rich blood from the gut through portal veins, the liver also directly contacts translocating bacteria and their various components and metabolites. Fortunately, in healthy settings, an intact and multilayered intestinal barrier restricts such direct host–microbiota contact and defends against excessive bacterial translocation. Another important interplay between the gut and the liver relies on bile acid metabolism. Synthesized in the liver and excreted into the gut along with other bioactive substances, primary bile acids are then converted into secondary bile acids by certain commensal microbes, especially *Clostridium* cluster XIV (1). About 95% of the bile acids are reabsorbed by the intestine, transported back to the liver, and secreted into the gut again, establishing a metabolic cycle called enterohepatic circulation. Within this cycle, bile acids modulate the composition of gut microbiota *via* selective pressure and, simultaneously, influence the metabolism and functionality of the liver. In addition to bile acids, many other host–microbiota–derived metabolites also take part in the bidirectional regulation utilizing similar routes, such as free fatty acids, choline, and ethanol derivatives (2). Through these complex interregulations, commensal bacteria and their metabolites help to maintain the metabolic and immune homeostasis of the liver. For instance, *Akkermansia muciniphila*, a Gram-negative anaerobic bacterium colonizing the mucus layer of the intestine, helps to alleviate intestinal inflammation and mitigate alcoholic and nonalcoholic liver damage (3, 4). Bile acids produced by certain bacteria can activate intestinal farnesoid X receptor (FXR) and thus promote fatty acid oxidation while reducing lipogenesis and lipid absorption in the liver, ameliorating hepatic inflammation and steatosis (5, 6). Another bacteria-derived metabolite, butyrate, helps to maintain gut barrier integrity and alleviate ethanol-induced liver injury (7). In short, the bidirectional communication between the host and the microbiota is essential not just to the health of the gut but also to that of the liver and probably the whole system.

Liver cirrhosis is a huge burden on public health worldwide. About one million deaths around the world are attributable to liver cirrhosis annually, making it the 11th most common cause of death and the third leading cause of death in people aged 45–64 years (8). According to WHO’s Global Burden of Diseases studies for 2019, liver cirrhosis was responsible for 560.4 age-standardized disability-adjusted life-years (DALYs) per 100,000 population globally, while liver cancer causes only 151.1 DALYs (9). The etiology of cirrhosis is rather complicated since various chronic liver diseases can lead to shrinkage of the liver parenchyma and overproduction of scar tissue. The most dominant causes of liver cirrhosis are hepatitis B and C, alcoholic liver disease (ALD), and nonalcoholic fatty liver disease (NAFLD) (8, 9). Despite the different pathological settings of these precirrhotic diseases, the trajectory of cirrhosis comes down to similar complications that mark the transition from compensated to decompensated cirrhosis. In a traditional perspective, variceal bleeding, ascites, and hepatic encephalopathy are the three major complications of cirrhosis, which result from portal hypertension, arterial vasodilation, and hyperammonemia, respectively. However, giving each complication or organ failure an independent pathophysiological mechanism does not seem to explain the complexity of decompensated cirrhosis well enough. Recent studies suggest that systemic inflammation and organ immunopathology are additional contributors to organ dysfunction, further verifying the notion that cirrhosis is a systemic disease (10, 11). For patients with acute decompensation of cirrhosis, the severity of systemic inflammation increases in parallel with the disease progression and the number of organ failures (10, 12, 13). Moreover, the significant correlations between systemic inflammation and portal hypertension, ascites, and hepatic encephalopathy indicate that systemic inflammation is the common pathophysiological mechanism for different complications of decompensated cirrhosis (13). Interestingly, gut microbes have long been considered the major source of systemic inflammation in cirrhosis (11, 13). Evidence indicates that gut dysbiosis is associated with the pathogenesis and progression of many precirrhotic diseases such as viral hepatitis, NAFLD, and ALD (14–19). Hepatic cirrhosis, as the advanced stage of these liver diseases, is linked to the altered composition and reduced diversity of gut microbiota despite etiology. Moreover, patients with cirrhotic conditions are prone to an impaired intestine barrier, pathological bacterial translocation, and systematic inflammation (20). Pathogen-associated molecular patterns (PAMPs) such as lipopolysaccharide (LPS) can stimulate immune cells and cytokine secretion in a Toll-like receptor (TLR)-NF-κB-dependent way, generating a proinflammatory and profibrogenic immune environment. Additionally, a bacterial infection is now regarded as the fourth major complication of decompensated cirrhosis because of its astonishingly high prevalence (21). The most common infection for cirrhosis patients, spontaneous bacterial peritonitis (SBP), is a perfect demonstration of how bacterial translocation constantly elicits inflammation and alters the host immunity (22). Therefore, it is tempting to speculate that gut microbiota contribute to the progression of hepatic cirrhosis through immune remodeling in a dysbiotic setting. Since bacteria are the most well-studied members of the gut microbiota and probably play a central role in microbiota–host interaction, this review will focus on gut bacteria but not viruses, fungi, archaea, or other microbes.

## Gut–liver axis contributes to immune homeostasis

### Gut homeostasis

The human intestine contains 10^14^ microbes, over 99% of which are bacteria. With such a huge quantity of microorganisms living inside the intestinal lumen, our system needs a strong defense to protect us from the excessive input of viable bacteria and their toxic metabolites. The multilayered intestinal barrier may serve as the first line of defense. The inner surface of the intestine is covered with a mucus layer that physically separates the bacteria from the intestine wall and delivers tolerogenic signals (23, 24). Beneath the mucus layer lies the intestinal epithelium, which consists of enterocytes, goblet cells, tuft cells, Paneth cells, M cells, and different immune cells (20). Adjacent epithelial cells form junctional complexes between each other to limit paracellular trafficking of intestinal contents. These complexes consist of desmosomes, adherens junctions (AJs), and tight junctions (TJs) (25). Paneth cells secrete α-defensins, islet-derived protein III-gamma (RegIIIγ), and lysozyme to defend against pathogens (26). Intraepithelial lymphocytes, including αβT cells and γδT cells, are activated by various cellular or cytokine signals to battle bacterial infection (20). Mononuclear phagocytes such as dendritic cells (DCs), with their processes sticking into the intestinal lumen, take part in both antibacterial immunity and oral tolerance (27). In the lamina propria, plasma cells secrete sIgA into the mucus layer to reinforce the frontline defense, while Th17 cells help to strengthen the tight junctions and promote epithelial regeneration. Microbial signals sensed by DCs or group 3 innate lymphoid cells (ILC3) trigger the secretion of IL-17 and IL-22, which promote the release of antibacterial peptides, mucin, and sIgA by other cells (28, 29). The last and most critical defense of the intestinal barrier, the gut–vascular barrier, is composed of endothelial cells, pericytes, and enteric glial cells. This gut–vascular unit is also reinforced by junctional complexes, allowing antigens from food or commensal bacteria to pass for tolerance induction but not bacterial translocation (20, 23).

**Figure 1:**
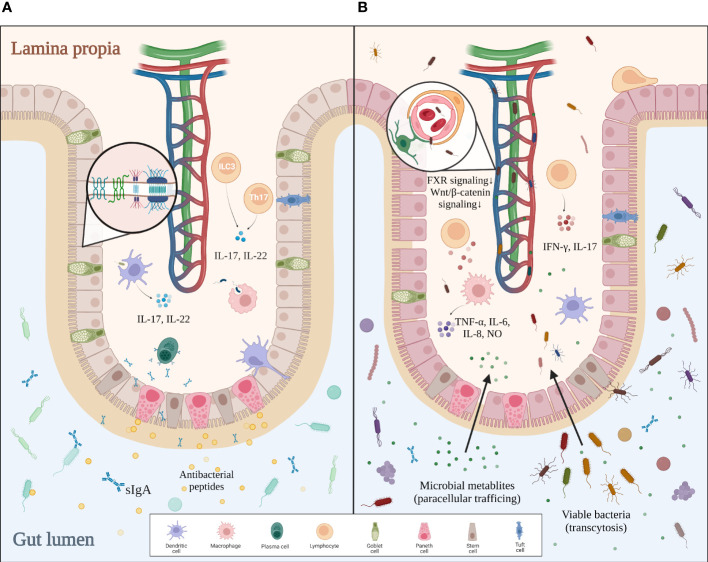
Comparison between intact and impaired gut barriers. **(A)** In healthy settings, the mucus layer serves as the first defense of the intestinal barrier against intraluminal bacteria. Epithelial cells tightly jointed together by junctional complexes limit the translocation of bacteria. Immune cells within the lamina propia not only actively remove invading pathogens by phagocytosis but also strengthen the gut barrier by secreting certain cytokines. Altogether, an intact gut barrier prevents pathological bacterial translocation. **(B)** In gut dysbiosis, bacterial overgrowth and disproportion can be found in the intestinal lumen. The mucus layer becomes thinner and looser, allowing pathogens to reach the epithelium. Disrupted junctional complexes and impaired gut–vascular barrier further promote pathological translocation. Additionally, dysregulated intestinal immunity aggravates inflammation and enterocyte injury, which eventually leads to a leaky gut.

Interestingly, this host–microbiota regulation is reciprocal, with recent studies proving that the intestinal barrier can be modulated by the gut microbiota. For instance, adhesion of certain microbes like segmented filamentous bacteria to the intestinal epithelial cells triggers robust induction of Th17 cells (30). When bacteria penetrate the inner layer of mucus, a group of sentinel goblet cells can nonspecifically sense microbial molecules and secrete more Muc2 mucin to expel the pathogens by activating the NLRP6 inflammasome downstream of ROS synthesis (31). CX3CR1^+^ macrophages are localized around the intestinal lamina propria vasculature, forming an interdigitating network to defend against pathogens. The conversion of such macrophages from CCR2^hi^CX3CR1^+^ monocytes is mediated by the microbiota in a Nr4a1-dependent manner (32). Furthermore, innate lymphoid cells in the lamina propria can maintain long-term antibacterial activity after being trained with bacteria (33). Bacteria-derived metabolites also contribute to the homeostasis of the gut. Indole is a tryptophan derivative produced by commensal bacteria that regulate the IL-22 expression of ILC3 *via* aryl hydrocarbon receptor (AHR). IL-22, in turn, modulates the secretion of antibacterial RegIIIγ by Paneth cells, making indole a favorable signal to gut homeostasis (34). Other studies suggest that indole reinforces the gut barrier integrity by increasing TJ resistance (35). To conclude, the host defense does not develop all by itself but rather depends on the constant stimulations from commensal microbes. In turn, active surveillance by intestinal immunity keeps gut microbes in line. Such delicate mechanisms of bilateral regulation guarantee a balance between tolerance for autochthonous microbes and antibacterial activities against pathogens in the gut **(** **Figure 1A** **)**.

### Liver homeostasis

The liver faces bacterial challenges constantly due to the unique anatomical and hemodynamic features of the portal system. If potent antibacterial immunity were induced each time the bacterial antigens reached the liver, there would be relentless inflammation and severe collateral damages to the system. Therefore, it is essential that liver parenchymal cells and other liver-resident cells form a fine-tuned immune network together and respond to these challenges in a well-balanced way.

Upon stimulation by gut-derived microbes, hepatocytes not only can secrete acute-phase proteins, complement proteins, and other bioactive substances to battle bacterial infection but also can play an important role in immune surveillance *via* expressing MHC I/II and costimulatory molecules. Liver sinusoidal endothelial cells (LSECs) allow the interaction between gut-derived molecules and the underlying hepatocytes and nonparenchymal cells *via* the special fenestrae (36). In addition to recruiting monocytes and lymphocytes in an ICAM-, VCAM-, or VAP-dependent manner, LSECs actively regulate the periportal distributions of myeloid and lymphoid cells *via* MYD88 signaling induced by gut commensal bacteria, resulting in more efficient prevention of systemic bacterial dissemination (37). Kupffer cells (KCs), a group of liver-resident macrophages that patrol the sinusoidal lumen, are important immune sentinels to detect, capture, and present bacterial antigens (38). Furthermore, KCs also regulate iron metabolism and prevent accumulative toxicity by removing damaged RBCs and hemoglobin from the bloodstream (39). Hepatic stellate cells (HSCs) crawl around the liver vasculature and store lipids and vitamin A in basal conditions. Whereas in the inflamed liver, HSCs transdifferentiate into fibrinogenic and immunomodulatory cells (40). Even though these different kinds of cells all express MHC and other antigen-presenting molecules, they are poor activators for T cells under most circumstances. Constant exposure to low-level LPS induces a refractory response in APCs, a phenomenon called endotoxin tolerance (41) and downregulation of costimulatory signals and upregulation of coinhibitory molecules like PD-L1 (38, 42). These alternations promote the development of regulatory T cells and cause incomplete activation, clonal anergy, and even premature death to the CD8^+^ T cells. In addition to direct contact, these antigen-presenting cells can also secrete inhibitory cytokines such as IL-10 and TGF-β, which dampen the activation and functions of CD4^+^ T cells and CD8^+^ T cells. Other studies suggest that Kupffer cells and HSCs prime the naïve CD4+ T cells toward Treg phenotype *via* secretion of prostaglandins (PGE2) and retinoic acid, respectively (38, 43). Furthermore, low-level LPS and proinflammatory cytokines like IFN-γ can induce the expression of indoleamine-2,3-dioxygenase (IDO) in Kupffer cells and DCs (42). This enzyme produces an immunosuppressive metabolite called kynurenine and contributes to the suppression of T-cell functions.

**Figure 2:**
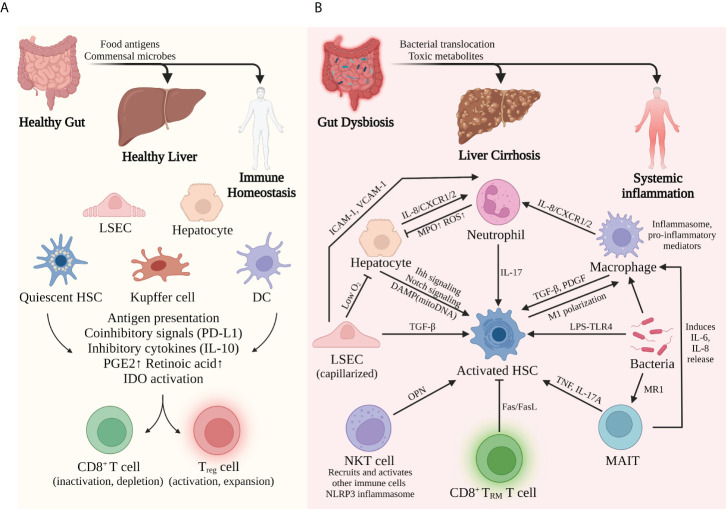
Liver immune environment in tolerogenic and immunogenic conditions. **(A)** In basal conditions, commensal bacteria and food antigens from a healthy gut help to maintain liver immune homeostasis. Constant exposure to LPS induces tolerance of APCs and therefore inactivation of CD8^+^ T cells but activation of T_reg_ cells. **(B)** By contrast, bacterial dysbiosis and impaired gut barrier promote the pathological translocation of viable bacteria and their products, causing massive inflammation in the liver and the whole system. The complicated interplay among different immune cells facilitates the proinflammatory and profibrogenic transformation of the liver. Activation of hepatic stellate cells seems to be the common mechanism for this transformation.

To conclude, gut-derived signals help to direct liver immunity to a tolerogenic phenotype that prevents immune overreaction in basal conditions. However, such immune tolerance does not exist without limitations. The liver still needs to efficiently mobilize immune cells and initiate an antibacterial response when a dangerous infection occurs. In fact, different receptors expressed by liver cells can distinguish antigens from commensal flora and those from pathogens. When excessive or dangerous signals are detected, APCs, including hepatocytes, LSECs, DCs, and Kupffer cells, can recruit neutrophils, natural killer cells, and lymphocytes to eliminate pathogens. In short, with the help of gut-derived signals, liver parenchymal and nonparenchymal cells construct a harmoniously coordinated network to maintain immune homeostasis **(** **Figure 2A** **)**.

## Dysfunctional gut–liver axis in liver cirrhosis

### Gut dysbiosis is related to liver cirrhosis

A diverse and relatively stable gut microbiome is essential to maintain the immune homeostasis of the host. Once initiated by certain insulting etiologies, gut dysbiosis slowly develops at the early stage of liver disease and progresses as the disease progresses. Researchers have confirmed that gut dysbiosis is closely associated with the pathogenesis and progression of hepatic cirrhosis. To begin with, reduced overall gene richness was found in the stool samples of cirrhotic patients (44, 45). Furthermore, a decreased α-diversity of gut microbiota is another feature of cirrhotic patients (46–48). β-Diversity was also significantly altered in individuals with NAFLD-cirrhosis (48, 49). These findings all suggest a less diverse and less stable gut microbiome in cirrhotic patients. Meanwhile, the composition of the gut microbiota is also changed adversely. Firmicutes and Bacteroidetes are the most dominant phyla of the human gut microbiota, followed by Proteobacteria and Actinobacteria with much smaller abundances. In cirrhotic settings, Proteobacteria and Fusobacteria are enriched while Bacteroidetes are depleted (44, 49–51). At the family level, potentially beneficial autochthonous taxa like *Lachnospiraceae*, *Ruminococcaceae*, and *Clostridiales XIV* are reduced while potentially pathogenic taxa including *Staphylococcaceae*, *Enterobacteriaceae*, and *Enterococcaceae* are increased (52). At the genus level, cirrhotic patients display higher abundances of buccal microbes including *Veillonella*, *Streptococcus*, and *Prevotella*, indicating that oral commensals may invade the intestine in cirrhotic conditions (44–48). At the species level, potential pathogens like *Ruminococcus gnavus*, *Veillonella parvula*, and *Streptococcus parasanguinis* are enriched, while beneficial commensals like *Eubacterium rectale* and *Faecalibacterium prausnitzii* are depleted (46, 48). Another marked alternation of the gut microbiota in cirrhotic patients is the small intestinal bacterial overgrowth (SIBO), partly due to decreased bowel motility, delayed transit time, and use of acid inhibitors and antibiotics (53, 54). Instead of being exclusive to cirrhosis, SIBO appears to be prevalent in different precirrhotic diseases, indicating that it develops as the disease progresses (55, 56) and correlates with disease severity (57). Even though alcohol consumption, a high-fat diet, virus infection, autoimmunity, and other cirrhosis-related factors may all affect the gut microbiota in certain ways, cirrhotic patients share similar microbiota profiles despite etiology. This might suggest that gut dysbiosis not only has something to do with the unique pathophysiological changes of cirrhosis but also partly results from some common medical interventions for cirrhosis. One possible explanation is that impaired hepatic function changes the intestinal environment (e.g., reduced bile flow and complement synthesis), which is then aggravated by cirrhosis complications (e.g., impaired gut motility due to ascites) and medical interventions (e.g., proton-pump inhibitors for variceal hemorrhage prevention). Further investigation is needed to figure out how gut dysbiosis initiates in cirrhosis patients.

### Impaired gut barrier promotes bacterial translocation and inflammation

Bacterial translocation (BT) is defined as the translocation of bacteria and/or bacterial metabolites from the gut lumen to mesenteric lymph nodes or the portal bloodstream. Though BT exists in basal conditions and helps to build tolerance for commensal microbes, its quantity markedly increases in pathological settings, eliciting a proinflammatory response and even systemic infection. As discussed before, an intact intestinal barrier is essential to prevent pathological bacterial translocation and to maintain immune homeostasis. This line of defense no longer holds in cirrhotic settings, with evidence showing the structural and functional breakdown of the gut barrier in cirrhosis (58, 59). Firstly, the mucus layer becomes thinner and easier for bacteria to colonize, even in the relatively denser and supposedly sterile inner layer. Other structural distortions include enlarged interepithelial space, shortening and widening of microvilli, submucosal edema, and disorganization of interepithelial TJs (20, 53, 58). In fact, these structural changes are related to decreased expression of tight-junction proteins occludin and claudin-1 (60, 61). Also, evidence has proven that the impairment of epithelial TJs is related to dysregulated fermentation of the gut microbiota. Ethanol and its toxic derivative acetaldehyde can damage the TJs directly and increase gut permeability in ALD (61, 62). The reduction of butyrate and other protective metabolites also contributes to the damaged barrier (7, 63). It is thought that weakened TJs promote the paracellular trafficking of bacterial metabolites, while translocation of viable bacteria likely depends on transcytosis (27, 54, 64). Although research on the detailed mechanisms of bacterial transcytosis is still lacking, many lines of evidence suggest that intestinal immune dysregulation induced by dysbiosis contributes to the bacterial penetration of the gut barrier. Cirrhotic rat models exhibit an inflammatory pattern of immune dysregulation in intraepithelial lymphocyte (IEL) and lamina propria lymphocyte (LPL), with an increase in activated lymphocytes and IFN-γ and IL-17 production (58, 65). Furthermore, dysbiosis-induced inflammation impairs the gut barrier *via* TNF-α/TNFR1 signaling mediated by monocytes and macrophages (62). Another study on cirrhosis patients also reveals that activated macrophages secrete NO, IL-6, and IL-8 that undermine the gut barrier, most probably under bacterial stimulation (59). By contrast, reduced synthesis and release of defensin, RegIIIβ/γ, and sIgA suggest impaired antibacterial functions of Paneth cells, neutrophils, B cells, and other epithelial cells (66). These findings are in line with a report of B-cell dysfunction in cirrhosis patients (67). Intestinal DCs also show a less-activated phenotype with decreased TNF-α production, deficient phagocytosis, and impaired migration in cirrhotic rats with excessive bacterial translocation (68). As for the final defense of the intestinal barrier, the gut vascular barrier is also damaged by certain pathogens such as *Salmonella typhimurium via* dampened β-catenin-dependent signaling (69). In dysbiotic conditions, reduced FXR signaling due to dysregulation of bile acid metabolism also impairs the integrity of GVB (70). To conclude, gut dysbiosis leads to the accumulation of invasive pathogens and toxic metabolites, which directly impair the gut barrier and cause intestinal inflammation. Local inflammation not only damages enterocytes but also weakens the antibacterial ability of the gut barrier. Altogether, these changes facilitate pathological bacterial translocation **(** **Figure 1B** **)**.

Translocated bacteria and gut-derived metabolites can directly interact with host cells. One of the most well-established mechanisms involves a group of receptors named pattern recognition receptors (PRRs), which are widely expressed on the surface of various hepatic and intestinal cells (38). PRRs include TLRs, nucleotide-binding oligomerization domain-like receptors (NLRs), C-type lectin receptors (CLRs), retinoic acid-inducible gene I-like receptors (RLRs), and absent in melanoma-2 (AIM2)-like receptors (ALRs) (71). Different PRRs can recognize different conserved molecular patterns of microbes (PAMPs) or damaged cells [damage-associated molecular patterns (DAMPs)]. For instance, a cell wall component of Gram negative bacteria, is a typical PAMP, while mitochondrial DNA released from injured cells belongs to DAMPs. Among these receptor–ligand interactions, TLR4-LPS is one of the most thoroughly studied and relevant pairs in cirrhosis. Upon recognition of LPS, TLR4 initiates downstream activation in both a MyD88-dependent manner and a TRIF-dependent manner. For MyD88-dependent signaling, TLR4-LPS interaction activates the MyD88-NF-κB pathway and leads to the production of proinflammatory cytokines such as TNF, IL-1, IL-6, and chemokines. For TRIF-dependent signaling (or MyD88-independent signaling), the TRIF-TBK1-IRF-3 axis is activated to secrete type I IFN (72). In short, interactions between PRRs and PAMPs/DAMPs promote the clearance of pathogens or damaged cells, thus causing inflammation-related damages in the gut and the liver in a cirrhotic setting.

### Gut-derived signals lead to an inflamed liver

In acute or chronic liver injury, especially liver cirrhosis, the delicate balance between tolerogenic and immunogenic responses is broken. Detrimental effects caused by an impaired gut barrier, bacterial translocation, alcohol consumption, and an unhealthy diet all contribute to the immune malfunction of the liver through the gut–liver axis. Chronic liver diseases such as ALD and NAFLD not only directly affect liver metabolism and immunity but also indirectly impaired liver function *via* a dysregulated intestinal barrier and gut dysbiosis. In response to these pathogenic conditions, hepatocytes and liver-resident immune cells may lose their normal functions and transform into proinflammatory, profibrogenic phenotypes that facilitate the progression of cirrhosis **(** **Figure 2B** **)**.

Hepatic stellate cells (HSCs) are the primary precursors for myofibroblasts during liver fibrosis (40). Gut-derived LPS can stimulate TLR4 of quiescent HSCs and activate these cells in a MyD88-NF-κB-dependent manner, thus causing profibrogenic transformation and accelerating liver fibrosis (73). Moreover, profibrogenic cytokines such as TGF-β and IL-17 are potent activators for HSCs and collagen production, which are excessively secreted by other hepatic cells under inflamed conditions (40, 74). A mouse model of liver fibrosis suggests that MyD88 signaling in activated HSCs promotes macrophage M1 polarization in a CXCL10/CXCR3-dependent manner, thus promoting liver fibrosis and inflammation (75).

Hepatocytes, the major parenchymal cells of the liver, play a crucial part in liver immune surveillance in health. However, recent studies suggest that hepatocytes might also promote liver cirrhosis in the presence of PAMPs and DAMPs. Activation of the TLR4-NF-κB pathway in hepatocytes promotes Jagged1/Notch signaling in the NASH mouse model, thus inducing OPN-dependent HSC activation and progressive fibrosis (76, 77). DAMPs released from injured hepatocyte mitochondria, mainly mtDNA, can directly activate HSCs and promote liver fibrosis. Such mito-DAMPs are increased in both mouse models and human patients with NASH and advanced fibrosis (78). In addition, TAZ is a transcription factor markedly elevated in the hepatocytes of human and murine NASH livers, which can initiate HSC activation in an Indian hedgehog (Ihh)-dependent manner and promote inflammation and fibrosis in NASH (79).

Neutrophils are recruited to the liver *via* the adhesion molecules ICAM-1 and VCAM-1 expressed by LSECs. These innate immune cells counteract bacterial infection mainly by phagocytosis and releasing lysozyme, ROS, elastase, and myeloperoxidase (MPO). Moreover, special extracellular fibrous structures named neutrophil extracellular traps (NETs) are formed to trap and eliminate pathogens (38). Studies suggest that neutrophils are involved in several cirrhosis-related conditions and the progression of cirrhosis (80). For example, nonalcoholic steatohepatitis (NASH) patients display significant neutrophil infiltration and activation in the liver. These cells secrete excessive MPO that directly kills hepatocytes, activates HSCs, and subsequently promotes liver fibrosis. Likewise, in cirrhosis, neutrophil functions are also disturbed by the dysfunctional gut–liver axis, leading to inflammation-related hepatocyte injury and IL-17-dependent HSC activation (81). Furthermore, intrahepatic neutrophils in patients with acute-on-chronic liver failure (ACLF) have higher expression of CXCR1 and CXCR2, receptors that are crucial for neutrophil recruitment, inflammatory mediator production (e.g., IL-8, IL-6, IL-23, CCL-20, and ROS), and contact-dependent cell death (82).

Hepatic macrophages can be divided into several subsets, among which Kupffer cells and monocyte-derived macrophages are essential players in maintaining immune homeostasis. In cirrhotic settings, especially ACLF, excessive bacterial translocation from the disrupted intestinal barrier exhausts the scavenging ability of macrophages and causes type I IFN-mediated IL-10 expression, resulting in a high risk of bacterial infection for cirrhosis patients. These macrophages also express a high level of MER tyrosine kinase (MERTK), which dampens the response to PAMPs and therefore antibacterial activity (83, 84). In addition to impaired bacterial clearance, macrophages also contribute to cirrhosis progression *via* promoting inflammation and fibrogenesis. Upon recognition of DAMPs and PAMPs, activated macrophages secrete proinflammatory mediators such as TNF, IL-1β, IL-6, IL-8, and ROS and promote the activation and survival of HSCs and myofibroblasts *via* TGF-β1 and PDGF (84). Macrophage-derived inflammasome resulting from bacterial translocation and tissue damage also contributes to the inflammatory injury to the liver (85). Such a multiprotein complex can be activated by PAMPs, DAMPs, ROS, cholesterol crystals, and other PRR ligands (86). Activated inflammasome initiates caspase-1-dependent production of proinflammatory cytokines like IL-1β and IL-18, which subsequently enhance liver inflammation, fibrosis, and damage in ALD, NASH, and other settings of liver injury (87).

Normally, mucosal-associated invariant T cells (MAIT) are anti-infection effectors that can be activated by bacterial metabolites from the vitamin B_2_ biosynthesis pathway in an MR1-dependent way (88). On the other hand, chronic liver inflammation exerts deleterious effects on the disease progression. A recent study suggests that MAITs are enriched in the fibrotic septa of cirrhotic patients, making direct contact with fibrinogenic cells. *In vitro* experiments show that MAIT can enhance the proinflammatory properties of myofibroblasts and monocyte-derived macrophages, further promoting liver fibrosis progression (89). Such MAIT–myofibroblast interaction can be achieved *via* the secretion of TNF and IL-17A by MAIT.

LSECs, along with Kupffer cells, constitute the most powerful scavenging system in the liver. LSECs maintain immune homeostasis by inducing tolerance to harmless antigens from food or commensal bacteria and maintaining quiescence of HSC (90). However, in acute or chronic liver injury, LSECs undergo several aberrant alternations in terms of morphology and function that promote a proinflammatory, profibrogenic, and proapoptotic phenotype. One of the major alternations in the capillarization of LSECs is a transformation characterized by the loss of fenestrae and the development of the basal membrane. Capillarized LSECs can no longer provide enough oxygen for the underlying hepatocytes, causing cell apoptosis, necrosis, and eventually the release of DAMPs (91). DAMPs and LSEC-derived signals such as TGF-β activate the profibrogenic HSCs and promote the generation of collagen and the progression of liver fibrosis. Dysfunctional LSECs also gain sinusoidal vasoconstriction ability due to reduced eNOS activity and increased vasoconstrictors, causing detrimental changes in liver hemodynamics that favor the development of portal hypertension.

Upon stimulation of DAMPs such as HMGB1, liver DCs can be switched from a tolerogenic, IL-10-producing phenotype to an immunogenic and TNF-producing phenotype. In addition, these cells activate NK cells and prime T cells within portal tract-associated lymphoid tissues (PALTs), further facilitating the progression of inflammation and tissue injury in a fibrotic setting.

NKT cells, a group of innate lymphoid cells that recognize endogenous or exogenous glycolipid antigens in a CD1d-dependent manner, are important regulators of liver immunity. When activated by different antigens or cytokines, type I NKT cells can activate NK, DCs, B, and T cells and recruit neutrophils and monocytes to the liver, propagating the liver inflammation. A recent study suggests that NKT promotes inflammatory response with the engagement of NLRP3 inflammasome in a mouse model of liver fibrosis (92). Moreover, type I NKT cells contribute to liver fibrosis *via* activation of the Hedgehog pathway and HSCs *via* secretion of osteopontin (OPN) (73, 93, 94).

To conclude, the massive release of PAMPs and DAMPs due to the impaired gut barrier and subsequent inflammation completely transforms the liver’s immune landscape. Tolerogenic properties in healthy conditions are replaced by immunogenic and fibrinogenic properties in cirrhotic settings, which feature the expansion of proinflammatory cells and cytokines and the deposition of the extracellular matrix. It is apparent that activation of HSCs is the common and central mechanism of cirrhosis progression induced by different cells **(** **Figure 2B** **)**, which is not surprising due to the collagen-producing function of activated HSCs. Moreover, during this transforming process, innate immunity seems to play the leading part, while adaptive immunity shows some protective effects. In a murine NASH model, CD8^+^ tissue-resident memory T cells promote liver fibrosis resolution by inducing apoptosis of hepatic stellate cells in a Fas/FasL-dependent manner (95). However, the interplay between innate and adaptive immunity in the development of cirrhosis still needs in-depth investigation.

## Cirrhosis-associated immune dysfunction

Cirrhosis-associated immune dysfunction (CAID) is the complex manifestation of an impaired immune system in the cirrhotic setting, mainly characterized by systemic inflammation and immune deficiency. CAID includes two phenotypes: the low-grade systemic inflammatory phenotype found in patients with compensated cirrhosis or decompensation without organ failure and the high-grade systemic inflammatory phenotype found in patients with ACLF (96). Evidence suggests that CAID is closely related to gut dysbiosis and impaired intestinal barrier, raising the idea that a dysfunctional gut–liver axis affects not only the immune environment of the gut and the liver but also the systemic immune functions.

### Systemic inflammation

Impaired gut barrier and bacterial dysbiosis excessively increase the bacteria and their components or metabolites within the blood flow. These PAMPs bind to PRRs of different organs and tissues, causing massive release of proinflammatory cytokines and activation of various immune cells and inflammasomes. Moreover, detrimental elements like endotoxins, alcohol, cholesterol, ROS, and the inflammation they induce will cause liver cell damage and thus the release of DAMPs into circulation, which further exacerbates the systemic inflammation. In the meantime, impaired liver functions render insufficient albumin, a protein that is supposed to neutralize excessive PAMPs during systemic inflammation. Likewise, deficient production of anti-inflammatory cytokines such as IL-10 by monocytes and Kupffer cells dampens the immune tolerance and promotes inflammation (96). Furthermore, stimulation by certain bacteria, immunogenic cell death, and oxidative stress in combination with excessive protein-folding demand during severe inflammation finally overwhelms the processing abilities of the endoplasmic reticulum, eliciting the unfolded protein response (UFR). UFR per se is a source of inflammation *via* secreting proinflammatory cytokines such as IL-6 and TNF. In turn, circulating cytokines like IL-1, IL-6, IL-8, and TNF can activate UFR in the liver, making a positive feedback loop that amplifies the systemic and hepatic inflammation (97, 98).

### Immune deficiency

Prolonged inflammation causes damage not only to parenchymal cells but also to the circulating immune cells. Immune cells show upregulated markers of activation but hampered immune response (99). For instance, in cirrhosis without ACLF, CD14^+^CD16^+^ monocytes are enriched and express more HLA-DR and TNF, favoring a proinflammatory and profibrogenic phenotype. However, their functions, such as phagocytosis, chemotaxis, and T-cell activation, seem to be inhibited. When the disease progresses to ACLF, expression of HLA-DR and TNF by these cells markedly decreases, as anti-inflammatory cytokines such as IL-10 appear to increase (96, 100). Likewise, neutrophils experience impaired antibacterial activity in the progression of cirrhosis (101). Moreover, continuous activation renders lymphocytes susceptible to anergy, apoptosis, and exhaustion. IFN-γ produced by T cells decreases, while inhibitory signals such as PD-1 and TIM3 increase (102). Changes in liver structure and function also contribute to immune deficiency. Extracellular matrix deposition, sinusoidal capillarization, intrahepatic shunting, and loss of hepatocytes all hamper the immunosurveillance and pathogen clearance functions of the liver (96). A recent study suggests that IL-2, a proinflammatory cytokine secreted by various cells in response to bacterial invasion, can markedly impair follicular T helper cells and therefore hamper the humoral immunity in advanced cirrhosis (103). Immune efficiency worsens as cirrhosis progresses, making advanced patients, especially ACLF patients, susceptible to severe systemic infection (104, 105).

## Summary and future perspectives

Liver cirrhosis is the advanced stage of various liver diseases, characterized by its diffuse, fibrinogenic, and nodule-forming changes pathologically. Cirrhosis patients are prone to gut dysbiosis, an impaired intestinal barrier, pathological bacterial translocation, and severe inflammation and fibrosis in the liver. It is not surprising to see how gut dysbiosis directly affects the progression of liver cirrhosis, given the close relationship they have in terms of anatomy, physiology, and metabolism. However, seeing how a dysregulated gut–liver axis can elicit such massive immune alternations in the fibrotic liver is still intriguing. The TLR4–LPS interaction seems to initiate most of the immune transformations in this process. A high level of PAMPs breaks the immune tolerance in the liver and causes prolonged inflammation and massive tissue damage. DAMPs released from injured cells further amplify the inflammation not only in the liver but also in the whole system. Eventually, immune paralysis occurs when the immune system gets overwhelmed by intense and continuous activation. During disease progression, hepatocytes and various nonparenchymal cells experience drastic changes in terms of phenotype and function. Among these changes, activation of HSCs appears to be the center of fibrosis progression, which is one of the pathological features of cirrhosis. However, HSC is not the entire story, as mounting evidence indicates that complex interplays exist among different immune cells.

Of note, due to the complexity of etiology, differences between various types of cirrhosis, such as virus-related cirrhosis, alcohol-related cirrhosis, NAFLD-related cirrhosis, and autoimmune-related cirrhosis, are not discussed in this review. It is of great importance to know that the etiology per se might, along with gut dysbiosis, contribute to the disease progression. For instance, alcohol alone can disrupt the gut barrier and cause inflammatory injury to the liver. Therefore, it might work in synergy with gut dysbiosis to reshape the immune environment of the liver. Relevant studies were extensively reviewed elsewhere (16, 63, 106, 107).

Given the great impact of gut dysbiosis on liver immunity, it is tempting to design therapy for cirrhosis patients targeting the gut–liver-immune axis. For microbiota modulation, fecal microbiota transplantation (FMT) and prebiotics/probiotics are proven to have beneficial effects on cirrhosis patients (53, 108, 109). Most interestingly, bacteriophages targeting pathogens such as *Enterococcus faecalis* can ameliorate alcoholic liver injury in an animal model, indicating a novel strategy to modulate gut microbiota (110). For gut barrier restoration, FXR agonists appear to be a promising choice (5, 70) while nonselective B-blockers (NSBBs) can reduce SIBO, and therefore BT, probably by (111) improving gut motility. Additionally, there is also new progress in modulating the liver-related immune response. Targeting TLR4 signaling and other cirrhosis-related proinflammatory pathways might be of great therapeutic potential (112, 113).

Though we are starting to comprehend the role of the gut–liver axis in the pathogenesis and immune remodeling of liver cirrhosis, multiple research perspectives remain largely elusive. Firstly, although researchers have identified some bacterial species that are correlated with liver cirrhosis, very few studies have discovered the mechanistic links between these specific species and cirrhosis progression. In addition to the common PAMP–PRR interaction, other components and metabolites of these species may have their own unique ways of communicating with the host. Secondly, intercellular communications in cirrhosis settings deserve more attention. The single-cell transcriptomic analysis begins to show its advantages in dissecting the complicated crosstalk among different immune cells in chronic liver diseases (95, 114). In the foreseeable future, multiomics studies, including transcriptomics, metagenomics, and metabolomics, will continue to provide fresh insights into the intrahepatic immune environment and host–microbiota interaction (115, 116). Thirdly, immune remodeling through the gut–liver axis goes far beyond the gut and the liver. Cirrhosis-related changes in other immune compartments such as the peritoneal cavity (22), lung, kidney, and brain (23) still warrant thorough investigations. Given the fact that gut-derived bacteria and metabolites are the major sources of systemic inflammation, a common condition associated with mortality for decompensated cirrhosis patients, it is very important to understand how the dysbiosis starts and how it affects the disease progression. Future studies need to focus on the cellular and molecular mechanisms of gut–liver–immune regulation and, hopefully, help patients benefit from these studies.

## Author contributions

Manuscript writing was done by HG. Manuscript revision was done by JY, MK, XZ, and HG. All authors contributed to the article and approved the submitted version.

## Funding

This project was supported by research funds from the RGC Theme-based Research Scheme (T12-703/19-R), the RGC Collaborative Research Fund (C7026-18G, C7065-18G), the Health and Medical Research Fund, Hong Kong (08191336), the National Natural Sciences Foundation of China (82103355) and CUHK direct grant.

## Acknowledgments

All figures were created with BioRender.com.

## Conflict of interest

The authors declare that the research was conducted in the absence of any commercial or financial relationships that could be construed as a potential conflict of interest.

## Publisher’s note

All claims expressed in this article are solely those of the authors and do not necessarily represent those of their affiliated organizations, or those of the publisher, the editors and the reviewers. Any product that may be evaluated in this article, or claim that may be made by its manufacturer, is not guaranteed or endorsed by the publisher.
